# Identifying Alzheimer’s disease and mild cognitive impairment with atlas-based multi-modal metrics

**DOI:** 10.3389/fnagi.2023.1212275

**Published:** 2023-08-31

**Authors:** Zhuqing Long, Jie Li, Jianghua Fan, Bo Li, Yukeng Du, Shuang Qiu, Jichang Miao, Jian Chen, Juanwu Yin, Bin Jing

**Affiliations:** ^1^Medical Apparatus and Equipment Deployment, Hunan Children’s Hospital, Changsha, Hunan Province, China; ^2^School of Biomedical Engineering, Capital Medical University, Beijing, China; ^3^Department of Pediatric Emergency Center, Hunan Children’s Hospital, Changsha, Hunan Province, China; ^4^Department of Traditional Chinese Medicine, Beijing Chest Hospital, Capital Medical University, Beijing Tuberculosis and Thoracic Tumor Research Institute, Beijing, China; ^5^Department of Medical Devices, Nanfang Hospital, Guangzhou, China; ^6^School of Electronic, Electrical Engineering and Physics, Fujian University of Technology, Fuzhou, Fujian, China; ^7^Beijing Key Laboratory of Fundamental Research on Biomechanics in Clinical Application, Beijing, China

**Keywords:** multi-modal imaging, brain atlas, Hurst exponent, support vector machine, artificial neural network

## Abstract

**Introduction:**

Multi-modal neuroimaging metrics in combination with advanced machine learning techniques have attracted more and more attention for an effective multi-class identification of Alzheimer’s disease (AD), mild cognitive impairment (MCI) and health controls (HC) recently.

**Methods:**

In this paper, a total of 180 subjects consisting of 44 AD, 66 MCI and 58 HC subjects were enrolled, and the multi-modalities of the resting-state functional magnetic resonance imaging (rs-fMRI) and the structural MRI (sMRI) for all participants were obtained. Then, four kinds of metrics including the Hurst exponent (HE) metric and bilateral hippocampus seed independently based connectivity metrics generated from fMRI data, and the gray matter volume (GMV) metric obtained from sMRI data, were calculated and extracted in each region of interest (ROI) based on a newly proposed automated anatomical Labeling (AAL3) atlas after data pre-processing. Next, these metrics were selected with a minimal redundancy maximal relevance (MRMR) method and a sequential feature collection (SFC) algorithm, and only a subset of optimal features were retained after this step. Finally, the support vector machine (SVM) based classification methods and artificial neural network (ANN) algorithm were utilized to identify the multi-class of AD, MCI and HC subjects in single modal and multi-modal metrics respectively, and a nested ten-fold cross-validation was utilized to estimate the final classification performance.

**Results:**

The results of the SVM and ANN based methods indicated the best accuracies of 80.36 and 74.40%, respectively, by utilizing all the multi-modal metrics, and the optimal accuracies for AD, MCI and HC were 79.55, 78.79 and 82.76%, respectively, in the SVM based method. In contrast, when using single modal metric, the SVM based method obtained a best accuracy of 72.62% with the HE metric, and the accuracies for AD, MCI and HC subjects were just 56.82, 80.30 and 75.86%, respectively. Moreover, the overlapping abnormal brain regions detected by multi-modal metrics were mainly located at posterior cingulate gyrus, superior frontal gyrus and cuneus.

**Conclusion:**

Taken together, the SVM based method with multi-modal metrics could provide effective diagnostic information for identifying AD, MCI and HC subjects.

## 1. Introduction

Alzheimer’s disease (AD), a progressive and irreversible neurodegenerative disease, is clinically characterized by a decline in cognitive, memory and learning, attention deficits, perceptual and visuospatial disability, and even ultimately death (Feng et al., 2019; Mao et al., 2021; Zhou et al., 2021), and the neuropathology of AD evolution is related to intracellular formation of neurofibrillary tangles consisted by tau-associated protein and extracellular deposition of abeta-associated amyloid (Desikan et al., 2009; Ota et al., 2014; Skolariki et al., 2020). Currently, approximately 40 million patients with AD exist worldwide, and the number is expected to increase threefold by 2050 (Kuang et al., 2021). Mild cognitive impairment (MCI), in which the cognitive function is mildly impaired compared to normal aging but does not reach to the criteria of senile dementia, is often considered to be a prodromal condition of AD (Raju et al., 2020), and 8–15% of patients with MCI would progress to AD per year (Kuang et al., 2021). As therapeutic interventions become available, developing valid multi-class discrimination methods for AD, MCI and health controls (HC) are desperately needed because effective clinical treatments for a progressive disease need to be adjusted according to different stages of the disease.

Multi-modal neuroimaging data such as functional magnetic resonance imaging (fMRI) and structural MRI (sMRI), in combination with advanced machine learning techniques, have attracted increasing attention from neuroradiologists, neuroscientists and neurologists in identifying AD, MCI and HC (Wang et al., 2019; Marin-Marin et al., 2021). The resting-state fMRI (rs-fMRI) based brain network analysis has been utilized as a promising technique in characterizing the abnormal topological organization in AD and MCI patients (Dyrba et al., 2015; Khazaee et al., 2016), and reduced intra-regional and inter-regional correlations of spontaneously brain activities in hippocampus or entorhinal cortex were detected in AD and MCI patients (Han et al., 2011; Ota et al., 2014). The sMRI offers quantification of brain tissue atrophy caused by cellular alterations underlying MCI and AD (Long et al., 2018a), and the atrophied brain regions were predominately involved in hippocampus, posterior cingulate cortex, and precuneus, etc. (Lei et al., 2020; Zhao et al., 2022). Given the consistent abnormal findings in the brain region of hippocampus from both fMRI and sMRI in many prior AD and MCI studies (Aguilar et al., 2013; Beheshti et al., 2016; Gonuguntla et al., 2022), we thus expect that the hippocampus based functional connectivity could provide some important information for AD and MCI identification. In addition, in combination with machine learning techniques, many prior studies demonstrated that the multi-modal neuroimaging data could considerably enhance the classification performance in AD or MCI discrimination because different modalities could offer complementary information to each other in comparison to single modality (Hojjati et al., 2018; Zhou et al., 2019a,b). However, another study suggested that the integration of different neuroimaging modalities did not improve the classification performance in identifying AD and MCI patients (Dyrba et al., 2015). These discrepancies indicate that the MCI and AD identification utilizing multi-modal imagings need to be further explored. Furthermore, to our best knowledge, only a few works have investigated the multi-class identification of AD, MCI and HC subjects simultaneously till now (Khazaee et al., 2016; Liu et al., 2018; Raju et al., 2020), and we speculate the multi-modal neuroimaging data could provide effective information for identifying multi-class of AD, MCI and HC subjects.

The brain consists of about 86 billion neural cells and a similar number of non-neural cells, which interacts within themselves or with others to form long-range or short-range connections, resulting in an interplay at different hierarchical temporal and spatial scales (Gentili et al., 2015). Interestingly, the connectivity properties of the neural networks have displayed associations with the spectrum profile of brain activities both in resting state and stimulating condition (Steinke and Galán, 2011). Also, the power spectrum of rs-fMRI signal has been shown a 1/f-like or fractal property (where f is frequency), and the power spectrum of fMRI signals can be expressed as with, which represents a modal of fractality (Gentili et al., 2015). The Hurst exponent (HE), an index ranging from 0 to 1, has a directly linear relationship with the parameter, which could also describe the fractal properties of fMRI signal well (Wei et al., 2013; Gentili et al., 2015). Actually, the time series could be classified into three categories based on its HE values. In detail, a HE equal or close to 0.5 indicates a random white noise; a HE smaller than 0.5 implies an anti-correlated time series, i.e., the fluctuation of time series is reversing in time; and a HE bigger than 0.5 suggests a correlated time series, i.e., the time series would go in the same direction along time (Wei et al., 2013; Gentili et al., 2015). Currently, the changes in HE of fMRI data have been investigated in autism disorder, major depressive disorder, MCI, normal aging and different personal traits (Maxim et al., 2005; Lai et al., 2010; Wei et al., 2013; Gentili et al., 2015). However, it is still unknown whether the HE of the fMRI data could be effectively combined with other characteristics for a multi-class classification of AD, MCI and HC.

Based on multi-modal metrics, we comparatively used two different machine learning methods including support vector machine (SVM) based classification methods and artificial neural network (ANN) to, respectively, perform a multi-class identification of AD, MCI and HC. In detail, the HE metric and the bilateral hippocampus independently based connectivity metrics generated from fMRI data, and the gray matter volume (GMV) metric obtained from sMRI were firstly calculated in each region of interest (ROI) based on a newly proposed Automated Anatomical Labeling (AAL3) atlas (Rolls et al., 2020). Then, these multi-modal metrics were selected with a minimal redundancy maximal relevance (MRMR) algorithm and a sequential feature collection (SFC) algorithm, and only a subset of the most discriminative features were retained after this step to construct the classification model. At last, the multi-class identifications of AD, MCI and HC subjects based on the constructed model were performed in single modality and multi-modal metrics respectively, and a nested ten-fold cross-validation was utilized to estimate the classification performance.

## 2. Materials and methods

### 2.1. Participants

A total of 180 participants including 49 AD, 69 MCI patients and 62 HC subjects were enrolled in this work, and all the participants were right-handed. All the AD and MCI patients were recruited from the memory clinic of the neurology department in Nanfang Hospital affiliated to Southern Medical University, and all HC subjects were recruited from local community by posting advertisement. All the participants did not take any medication that might affect cognitive functions during the scan, and these three groups were well matched in age, gender and education level. This study was authorized by the ethics committee of Nanfang Hospital, in accordance with the rules of the declaration of Helsinki, and written informed consents from all participants were obtained. Before the scan, all participants were estimated with a standardized clinical assessment protocol including clinical dementia rating (CDR) scale, mini-mental state exam (MMSE) and auditory verbal learning test (AVLT), and the diagnosis of AD, MCI and HC subjects were made by two experienced neurologists with the following criteria (Knopman et al., 2003; Li et al., 2017; Zhou, 2021). Of note, 5 AD, 3 MCI and 4 HC subjects were discarded from further analyses because of excessive head motion during the scan, and the detailed clinical characteristics and demographics for the remaining subjects were listed in Table 1.

**Table 1:** Participant demographic and clinical characteristics.

Characteristics	AD	MCI	HC	*p* values
Gender (M/F)	44(24/20)	66(30/36)	58(26/32)	0.56^a^
Age (years)	67.70 ± 5.24	67.11 ± 7.22	65.21 ± 7.42	0.14^b^
Education (years)	9.45 ± 4.72	9.74 ± 4.18	10.14 ± 4.33	0.73^b^
CDR	1	0.5	0	0^b^
MMSE	20.81 ± 2.25	24.96 ± 1.98	28.76 ± 1.05	<0.0001^b^
AVLT-ir	5.50 ± 3.46	7.95 ± 2.56	13.00 ± 2.98	<0.0001^b^
AVLT-dr	2.20 ± 2.55	4.15 ± 3.20	9.89 ± 2.72	<0.0001^b^
AVLT-r	4.45 ± 2.69	7.05 ± 3.51	11.53 ± 2.24	<0.0001^b^

a The *p* value was obtained by Chi-square test.

b The *p* values were obtained by the one-way analysis of variance.

Values are mean ± S.D unless the S.D was not calculated; M, male; F, female; CDR, Clinical Dementia Rating scale; MMSE, Mini-Mental State Examination; AVLT, Auditory Verbal Learning Test; AVLT-ir, AVLT-immediate recall; AVLT-dr, AVLT-delay recall; AVLT-r, AVLT-recognition.

AD criteria: (1) diagnosed with AD according to the DSM-IV criteria (Diagnostic and Statistical Manual of Mental Disorders, 4th edition, revised); (2) CDR score of 1.0; (3) able to carry out the clinical assessments and tolerate MRI scan.

MCI criteria: (1) CDR score of 0.5; (2) Not meeting the criteria of dementia according to DSM-IV; (3) objective memory complaints, adjusted for education and age; (4) normal or near normal activities of daily living; (5) normal or near normal performance of general cognitive function.

HC criteria: (1) CDR score of 0; (2) without memory complaints; (3) normal activities of daily living; (4) normal cognitive and physical status.

The exclusion criteria for all participants included: (1) depression, schizophrenia and other psychiatric diseases that can interfere with cognition functions; (2) epilepsy, brain tumors, encephalitis, Parkinson’s syndrome and other nervous system disorders that could result in cognitive function impairments; (3) severe brain injury that can influence cognitive functions; (4) those who have a history of congenital mental growth retardation, stroke or psychosis; (5) those who have a dependence of alcohol, (6) those who have contradictions for MRI examination or have visible vascular lesions on sMRI data.

### 2.2. Data acquisition

All the MRI images were obtained on a 3.0 Tesla Siemens scanner with an eight-channel radio frequency coil at Nanfang hospital. Two ear plugs were adopted to reduce the scanning noise, and a comfortable foam padding was utilized to restrict head motion. All participants were told to not move as long as possible, to open the eyes and to keep relax during the scan. The sMRI data were acquired using a magnetization prepared rapid gradient echo (MPRAGE) three-dimensional T1-weighted sequence with following parameters: repetition time (TR) = 1900 ms, inversion time (TI) = 900 ms, echo time (TE) = 2.2 ms, flip angle (FA) = 9 degree, matrix = 256 × 256, thickness = 1.0 mm, number of slice = 176, and voxel size = 1 × 1 × 1 mm^3^. The fMRI data were acquired with an echo-planar imaging (EPI) sequence with the following parameters: TR = 2000 ms, TE = 40 ms, FA = 90 degree, matrix = 64 × 64, thickness = 4 mm, number of slice = 28, field of view (FOV) = 240 × 240, and voxel size = 3.75 × 3.75 × 4 mm^3^.

### 2.3. Data pre-processing

#### 2.3.1. fMRI data

Data pre-processing for all the fMRI images were carried out by the toolbox of Data Processing Assistant for Resting-State fMRI (DPARSF) (Yan and Zang, 2010). Specifically, after the removal of the first 10 volumes for signal equilibrium, the remained 229 volumes were slice-time corrected and then realigned to the first volumes. A total of 12 subjects including 5 AD, 3 MCI and 4 HC subjects were excluded due to excessive head motion (2 mm and 2 degree in all direction). After that, the structural T1-weighted images for every subjects were co-registered to the fMRI data, and the co-registered structural images were then segmented and normalized to the Montreal Neurological Institute (MNI) space. Then, all realigned functional images were normalized to the MNI space by utilizing the parameters obtained from the structural data normalization, and all these images were resampled into a voxel size of 3 × 3 × 3 mm^3^. Next, nuisance covariates including the mean global signals, the 6 head-motion parameters, and the mean signals in white matter (WM) and cerebrospinal fluid (CSF) were regressed out from the normalized functional images. At last, all these normalized fMRI data were band-pass filtered (0.01–0.10 Hz) and spatially smoothed with a 4 mm full width at half maximum Gaussian kernel.

#### 2.3.2. sMRI data

Before pre-processing the sMRI images with the toolbox of voxel based morphometry (VBM) implemented in Statistical Parametric Mapping (SPM8), all the T1-weighted images were screened by two professional neuroradiologists with no obvious lesion or abnormality. Then, all these screened sMRI data were segmented into the gray matter (GM), WM and CSF by utilizing the routine of ‘New-segment’ within VBM. Next, these segmented images were normalized into the MNI space by using the diffeomorphic anatomic registration through exponentiated lie (DARTEL) approach. After this, the normalized images were modulated with the Jacobian metrics to preserve the actual amounts of a tissue class within each voxel. At last, an 8 mm full-width-half-maximum Gaussian kernel was adopted to smooth all the modulated images.

### 2.4. Feature extraction based on AAL3 atlas

**Figure 1:**
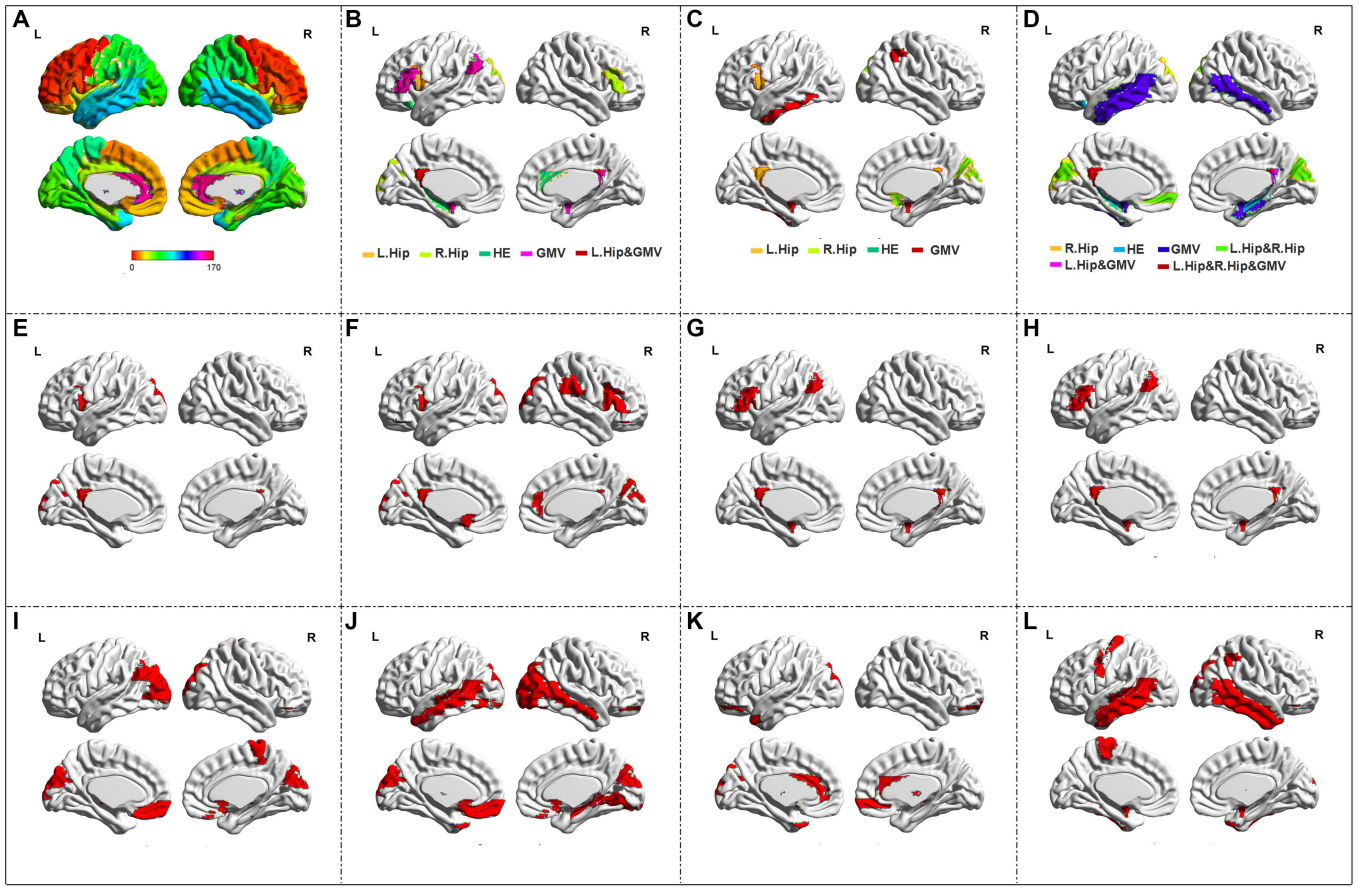
The automated anatomical Labeling (AAL3) atlas and the abnormal brain regions in single modality or multi-modal metrics. **(A)** The AAL3 atlas; **(B,C)** the abnormal regions in identifying the combined **A,D** and MCI from HC, and in identifying **A,D** from MCI in the SVM model with all metrics respectively; **(D)** the abnormal regions in the ANN model with all metrics; **(E-H)** the abnormal regions of L. Hip seed based connectivity, R. Hip seed based connectivity, HE and GMV in identifying the combined AD and MCI from HC in the SVM model respectively; **(I-L)** The abnormal regions of L. Hip seed based connectivity, R. Hip seed based connectivity, HE and GMV in identifying AD from MCI in the SVM model respectively.

Four kinds of imaging metrics including the functional HE characteristic and bilateral hippocampus seed independently based functional connectivity obtained from fMRI data, and the structural GMV obtained from sMRI data were achieved. In detail, the HE value of the fMRI in each voxel was calculated by the range scaled (R/S) analysis, and the detailed principles for the calculation of the HE value have been elaborately illustrated in several prior studies (Wei et al., 2013; Jing et al., 2017). In terms of hippocampus seed based function connectivity, bilateral hippocampus were independently adopted to serve as the seed to build the functional connectivity mapping, and the Pearson correlation coefficients between each voxel of the brain cortex and the hippocampus (numbers of 41–42 in AAL3 atlas) were computed. According to the partition criteria of the AAL3 atlas for the brain cortex (Figure 1A, available at: https://www.oxcns.org/aal3.html), the mean values of these functional mappings in each region of the AAL3 atlas were extracted, and the GMV values for all subjects in each region of the AAL3 atlas were also calculated by utilizing the following Matlab code.^1^ Actually, because two small regions (numbers of 133–134) were not defined after the original voxel size of 1 × 1 × 1 mm^3^ was resampled into 3 × 3 × 3 mm^3^, and four brain regions (numbers of 35–36, 81–82) remain empty in the AAL3 atlas, thus the number of utilized regions of the AAL3 atlas is 164 with the maximum label number of 170.

### 2.5. Feature selection

Effective feature selection algorithms are essential to machine learning methods, which can reduce the feature dimensionality and storage requirements, speed up the computation time, and improve the classification performance (Dai et al., 2012; Hojjati et al., 2018). In this work, the MRMR algorithm in combination with the SFC method were simultaneously used to select the optimal features for improving the multi-class identification of AD, MCI and HC subjects. The MRMR score for a feature set is estimated by the following formula:

(1) $$MRMR=MAX_{s}\left\{\frac{1}{\left|S\right|}\underset{f_{i}\in s}{\sum }I\left(f_{i},c\right)-\frac{1}{{\left|S\right|}^{2}}\underset{f_{i}\in s}{\sum }I\left(f_{i},f_{j}\right)\right\}$$

Where the relevance between a feature set *S* and the corresponding class *C* = {*c_1,_ c*_*2*,..._ *c_k_*} is estimated by the average value of all mutual information values between the individual feature *f_i_* and *C,* and the redundancy for a feature set *S* is defined by the mean value of all mutual information values between individual feature *f_i_* and *f_j_*. In this work, the prior 50 features were extracted by the MRMR algorithm firstly, and then these extracted features were utilized by the SFC algorithm to further perform different combinations of features for discriminating a multi-class of AD, MCI and HC subjects. In detail: In the first loop, the SFC method started with the top 2 features of sorted 50 features for estimating the performance of the multi-class identification, and then the number of features were increased one-by-one to perform the multi-class identification until all the 50 features were utilized. In the second loop, the SFC method discarded the first feature from the sorted 50 features, and then the multi-class identification was carried out repeatedly with the number of features ranging from the remained first 2 to the remained 49 features in a one-by-one growth pattern. As the top features were eliminated one-by-one in every loop, the multi-class identification was repeatedly performed until the loop number reached up to 49, which means that only the last 2 features of the sorted 50 features were remained for multi-class identification in the last loop. Lastly, the best optimal subset of features was determined by comparing the classification performance of all the subsets of features.

### 2.6. Classification

#### 2.6.1. SVM based multi-class identification

**Figure 2:**
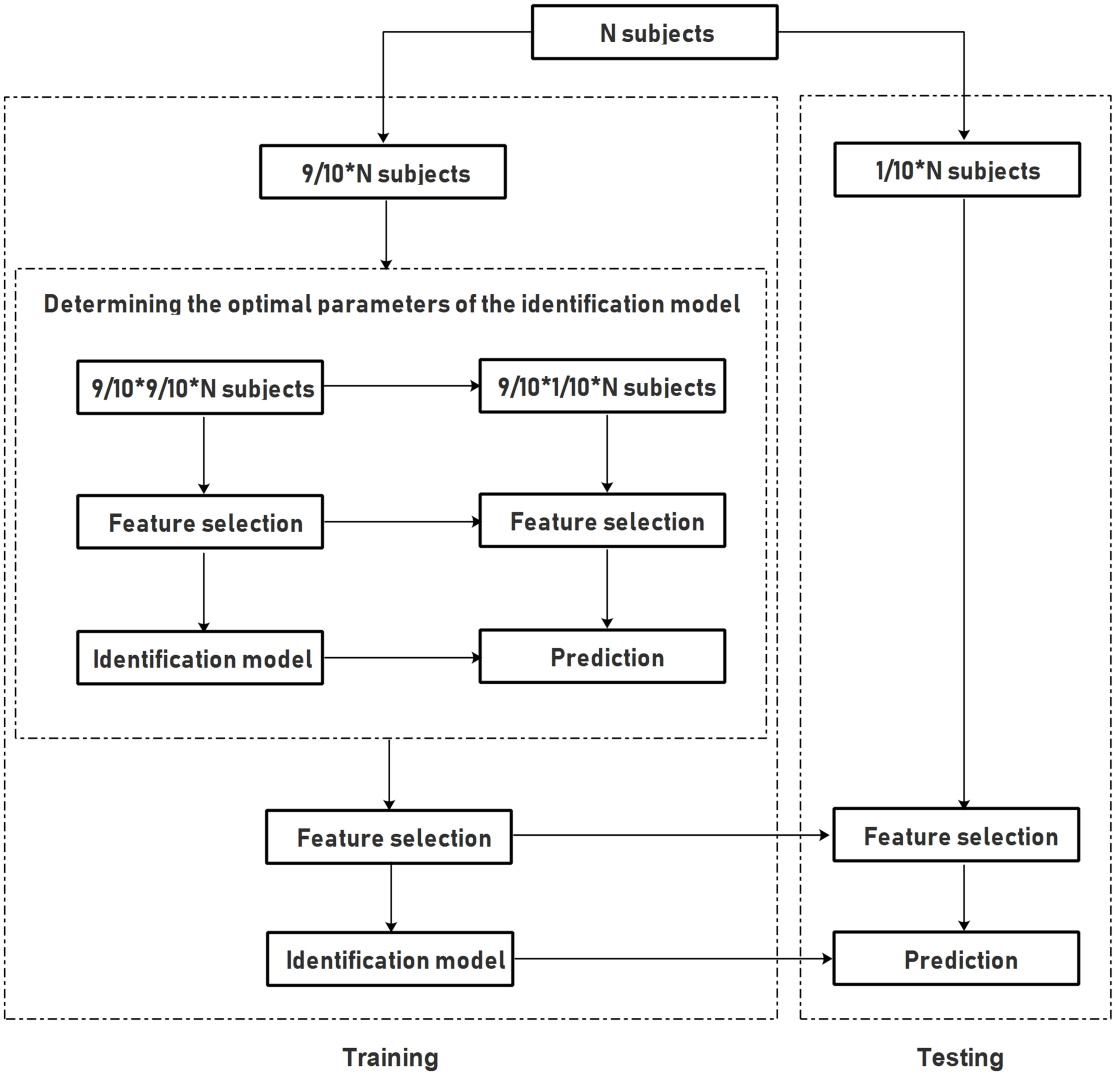
The framework of the nested 10-fold cross-validation.

The SVM method, which is widely utilized due to its simple theory and implementation as well as its remarkable power for classification, was originally developed for binary classification (Dyrba et al., 2015; Beheshti et al., 2016). To perform the multi-class identification of AD, MCI and HC, we therefore combined the AD patients with MCI patients to form a patient group firstly, and then the SVM method was utilized to perform the binary classification for identifying the mixed AD and MCI patients from HC subjects. Next, the SVM method was further performed for the correctly classified patients to discriminate AD from MCI patients. Through the above-mentioned processes, the classification accuracy in these three groups were evaluated. In particular, to improve the classification performance, the SVM method adopted a radial basis function (RBF) as the kernel function to deal with the nonlinear relationships between the labels and features, and the grid search method was utilized to optimize two parameters with the range of *C* = 2^−8^, 2^–7.5^, 2^−7^, ... 2^7.5^, 2^8^ and gamma = 2^−8^, 2^–7.5^, 2^−7^, ... 2^7.5^, 2^8^. Actually, these two parameters were optimized by an internal ten-fold cross-validation on the training data, and the overall of the nested ten-fold cross-validation classification framework were shown in Figure 2. In detail, all subjects were divided into 10 parts in the external ten-fold cross-validation. Within, nine parts of them were utilized as the training data to tune the classification model, and the remaining one part was used as the testing data to estimate the classification performance. These tuning and testing procedures were repeatedly 10 times so that each part of the data was utilized as the testing data once. In the internal ten-fold cross-validation, the training data was further divided into 10 subsets. Within, nine subsets were utilized to determine the optimal parameters of *C* and gamma, and the remaining one subset was used to test the performance of the selected parameters. The parameters with the best accuracy were adopted as the optimal parameters for classification model. Lastly, all the training data were utilized to construct the classification model with the optimal parameters, and the model performance was estimated by the testing data with an external ten-fold cross-validation.

#### 2.6.2. ANN based multi-class identification

The ANN algorithm, which was inspired from the attributes of biological nervous system that how the human brain adapts itself and learn, is useful for categorization and prediction (Sada and Ikpeseni, 2021), and the ANN model can be expressed with the following formula:

(2) $$f\left(x\right)=g\left(\underset{j}{\sum }w_{kj}g\left(\underset{i}{\sum }w_{ji}x_{i}\right)\right)$$

The ANN model is executed by iteratively adjusting the weights to capture the nonlinear or linear relationship between the input signals and outputs with an acceptable error limit (Qiu et al., 2021). In this work, the logistic function was adopted as the activation function (i.e., the function *g* in eq. (2)), and the root mean squared error (RMSE) was served as the statistical measure for estimating the neural network (RMSE <0.01 or maximum epoch = 500). Similar to the above-mentioned classification process, the nested ten-fold cross validation was also utilized for the ANN model to identify the multi-class of AD, MCI and HC. In detail, the external ten-fold validation cross was utilized to evaluate the final classification performance of the multi-class identification, and the internal ten-fold cross validation was utilized to determine the optimal number of neuron nodes in the middle layer with the range of 2 to 20. To make a better comparison with the SVM based model, the ANN model was set similarly as SVM model with two binary classifications using all MRI metrics for identifying AD, MCI and HC subjects.

### 2.7. Validation analysis

Validation analysis were focused on the influences of three potential factors in the classification models: (1) direct three-class identification; (2) unbalanced sample size; (3) atlas choice. To classify AD, MCI and HC subjects directly with a three-class identification is another recognition manner, therefore, we testify ANN as well as two additional algorithms including the naive Bayes and the random forest (RF) (Ma et al., 2020) on all subjects with all metrics, which were implemented using the Statistics and Machine Learning Toolbox in Matlab. Considering the uncertain impact of unbalanced sample size on model performance, subjects of all three groups were randomly down-sampled into the same size of 44 samples, and the SVM and ANN models were again tested with all metrics accordingly. Different brain atlases parcel the whole brain with distinct nodes, which may also affect the model performance. Consequently, another commonly used brain atlas named Brainnetome atlas, partitioning all the brain into 246 nodes including 210 cortical sub-regions and 36 subcortical sub-regions (Fan et al., 2016), was also adopted to compare the classification results obtained by the AAL3 atlas.

## 3. Results

### 3.1. Classification performance of different models

**Figure 3:**
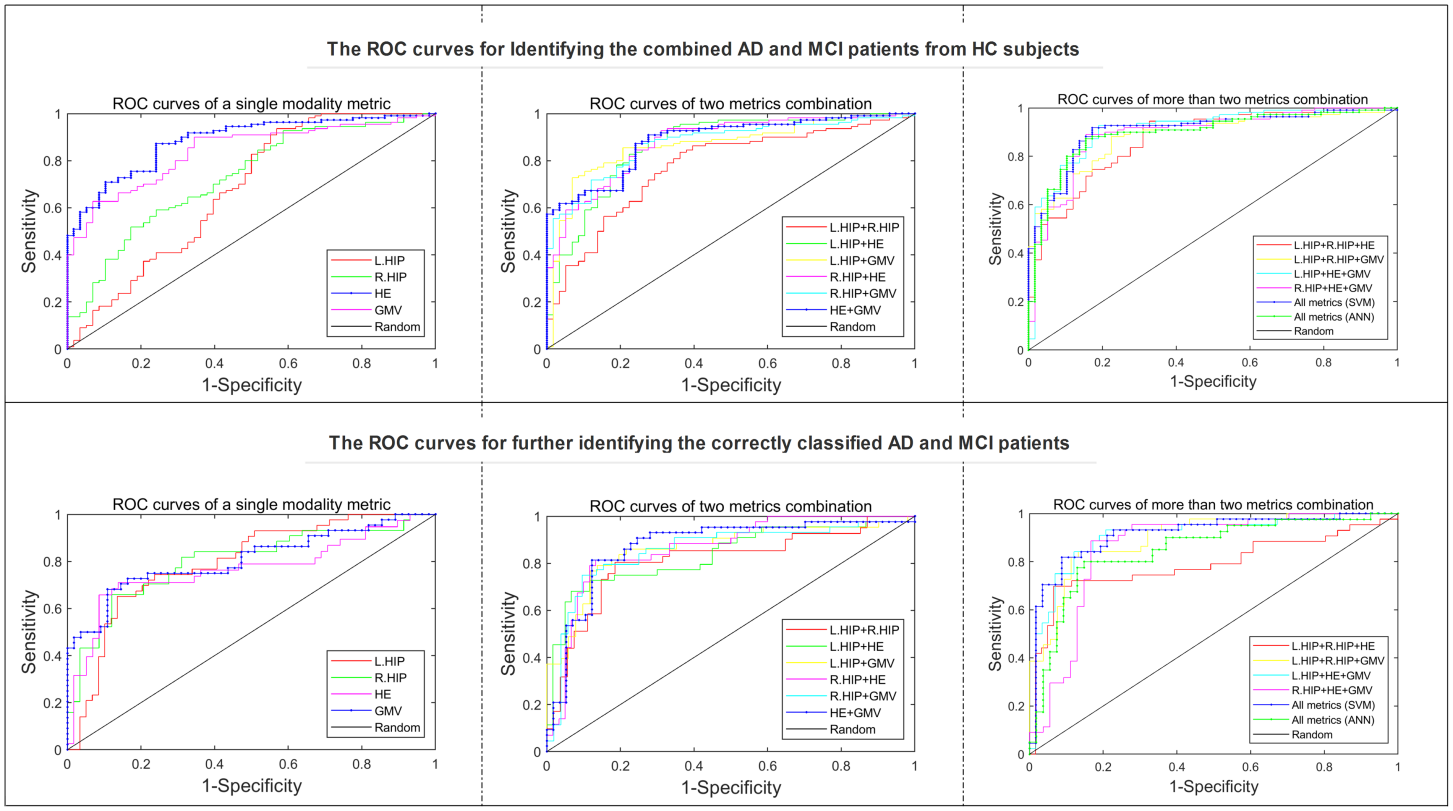
Two kinds of ROC curves for identifying AD, MCI and HC subjects.

By applying the SVM based method to identify the multi-class of AD, MCI patients and HC subjects, a best accuracy of 80.36% was obtained by utilizing all these four metrics simultaneously, and the correctly classified rates in AD, MCI and HC were 79.55, 78.79 and 82.76%, respectively. Besides, when adopting single modality metric, the SVM based method obtained a best accuracy of 72.62% with the HE metric, and the correct rates in AD, MCI and HC were 56.82, 80.30 and 75.86%, respectively. By applying the ANN method to classify these three groups with two binary classifications, a best accuracy of 74.40% was yielded by using all metrics, and the correct rates in AD, MCI and HC were 72.73, 66.67 and 84.48%, respectively. The detailed classification results of the SVM based method for all kinds of combinations of these four metrics and the results of two binary classifications of the ANN model with all metrics were displayed in Table 2. In addition, two kinds of receiver operating characteristic (ROC) curves including discriminating the combined AD and MCI from HC and the further discrimination between AD and MCI were shown in Figure 3, and the best area under curve (AUC) values of these two kinds of curves were the same 0.91 by using all metrics in the SVM models.

**Table 2:** The detailed results of the SVM based method for all kinds of combinations of four metrics and the ANN model with all metrics.

Metrics	AD vs. MCI vs. HC performance				(AD&MCI) vs. HC performance					AD vs. MCI performance					
	Overall	AD	MCI	HC	No. of features	Overall	AD&MCI	HC	AUC	No. samples (AD/MCI)	No. of features	Overall	AD	MCI	AUC
L.HIP	61.90	63.64	77.27	43.10	6	75.60	92.73	43.10	0.67	43/59	30	77.45	65.12	86.44	0.79
R.HIP	61.90	65.91	77.27	41.38	23	75.00	92.73	41.38	0.73	44/58	45	78.43	65.91	87.93	0.80
HE	72.62	56.82	80.30	75.86	39	83.33	87.27	75.86	0.89	38/58	29	81.25	65.79	91.38	0.77
GMV	69.64	68.18	74.24	65.52	8	81.55	90.00	65.52	0.84	44/55	28	79.80	68.18	89.09	0.81
L.HIP + R.HIP	66.67	75.00	66.67	60.34	13	77.38	86.36	60.34	0.77	41/54	6	81.05	80.49	81.48	0.81
L.HIP + HE	73.21	65.91	84.85	65.52	15	84.52	94.55	65.52	0.86	44/60	7	81.73	65.91	93.33	0.83
L.HIP + GMV	72.62	75.00	68.18	75.86	11	82.14	85.45	75.86	0.87	43/51	12	82.98	76.74	88.24	0.86
R.HIP + HE	73.21	70.45	81.82	65.52	23	83.93	93.64	65.52	0.88	43/60	9	82.52	72.09	90.00	0.86
R.HIP + GMV	73.21	72.73	71.21	75.86	5	83.33	87.27	75.86	0.87	44/52	10	82.29	72.73	90.38	0.85
HE + GMV	75.00	79.55	74.24	72.41	6	84.52	90.91	72.41	0.89	43/57	4	84.00	81.40	85.96	0.87
L.HIP + R.HIP + HE	75.00	68.18	86.36	67.24	34	85.12	94.55	67.24	0.87	43/61	7	83.65	69.77	93.44	0.79
L.HIP + R.HIP + GMV	76.19	81.82	71.21	77.59	26	84.52	88.18	77.59	0.88	44/53	21	85.57	81.82	88.68	0.90
L.HIP + HE + GMV	79.17	75.00	81.82	79.31	16	88.10	92.73	79.31	0.91	44/58	7	85.29	75.00	93.10	0.91
R.HIP + HE + GMV	79.17	88.64	68.18	84.48	22	87.50	89.09	84.48	0.89	44/54	15	85.71	88.64	83.33	0.86
L.HIP + R.HIP + HE + GMV (SVM)	80.36	79.55	78.79	82.76	17	88.69	91.82	82.76	0.91	44/57	15	86.14	79.55	91.23	0.91
L.HIP + R.HIP + HE + GMV (ANN)	74.40	72.73	66.67	84.48	21	85.12	85.45	84.48	0.90	40/54	18	80.85	80.00	81.48	0.85

L.Hip, left hippocampus seed based connectivity; R.Hip, right hippocampus seed based connectivity; HE; Hurst exponent; GMV, gray matter volume.

### 3.2. Characteristic brain abnormalities among three groups

The abnormal brain regions appeared no less than 6 times in the whole external 10-fold cross-validation with the optimal classification conditions in identifying the combined AD and MCI from HC, and in further identifying AD from MCI in the SVM model were shown in Figures 1B,C, respectively. The number of retained features and the abnormal brain regions in each fold of the external 10-fold cross-validation in the SVM model using all metrics were summarized in Supplementary Tables S1, 2. When using the ANN model to directly identify these three groups, the most abnormal brain regions were displayed in Figure 1D. The overlapping abnormal brain regions for these four metrics were mainly located at posterior cingulate gyrus, superior frontal gyrus and cuneus. In addition, when using the SVM model to identify the combined AD and MCI from HC with single metric, the abnormal brain regions of the left hippocampus (L.Hip) seed based connectivity, the right hippocampus (R.Hip) seed based connectivity, the HE and the GMV were detailedly displayed in Figures 1E–H, respectively. When using the SVM model to further identify the AD from MCI patients, the abnormal regions of the L.Hip seed based connectivity, the R.Hip seed based connectivity, the HE and the GMV were elaborately shown in Figures 1I–L, respectively. Besides, the abnormal regions of the single modality and all metrics based classification in the SVM models and all metrics based direct three-class identification in ANN model were summarized in the Table 3. At last, the results of the correlation analyses between the clinical assessments and the prior three features in each metric were shown in Table 4.

**Table 3:** The abnormal brain regions in single or all metrics based classification model.

Metrics	(AD&MCI) vs. HC	AD vs. MCI
L.Hip	Inferior frontal gyrus[7], Posterior cingulate gyrus[39], Superior occipital gyrus[53], Lobule I and II of vermis[113], Lobule X of vermis[120], Ventral tegmental area[159]	Superior frontal gyrus[21], Superior occipital gyrus[53], Lobule VIIB of cerebellar[105], Lobule X of vermis[120], Pulvinar lateral of thalamus[147], Raphe necleus, dorsal[169]
R.Hip	Inferior frontal gyrus[7], Olfactory cortex[17], Lateral orbital gyrus[31], Posterior cingulate gyrus[39], Superior occipital gyrus[53], Red nucleus[165]	Olfactory cortex[18], Cuneus[50], Lobule VIIB of cerebellar[106], Lobule I and II of vermis[113], Lobule X of vermis[120], Lateral posterior of thalamus[124]
HE	Anterior orbital gyrus[27], Posterior orbital gyrus[29], Hippocampus[41], Lobule IX of vermis[119], Lateral posterior of thalamus[123], Lobule I, II of vermis[113]	Anterior orbital gyrus[27], Lobule VIIB of cerebellar[105], Mediodorsal medial magnocellular of thalamus[136], Medial geniculate of thalamus[142], Pulvinar lateral of thalamus[148], Locus coeruleus[168]
GMV	Posterior cingulate gyrus[39], Amygdala[45], Amygdala[46], Angular gyrus[69], Petuman[77], Lobule X of cerebellar[112]	Amygdala[45], Amygdala[46], Inferior temporal gyrus[93], Lobule X of cerebellar[112], Lobule X of vermis[120], Ventral anterior of thalamus[126]
All metrics for SVM model	**L.Hip:** Inferior frontal gyrus[7], Posterior cingulate gyrus[39], Lobule I, II of vermis[113] **R.Hip:** Inferior frontal gyrus[10], Superior occipital gyrus[53] **HE:** Posterior orbital gyrus[29], Hippocampus[41], Anterior cingulate cortex[156] **GMV:** Posterior cingulate gyrus[39], Posterior cingulate gyrus[40], Amygdala[45], Amygdala[46], Angular gyrus[69], Putamen[77]	**L.Hip:** Inferior frontal gyrus[7], Posterior cingulate gyrus[39]; Lobule I, II of vermis[113] **R.Hip:** Olfactory cortex[18], Cuneus[50], Lateral posterior of thalamus[124] **HE:** Pulvinar lateral of thalamus[147], Pulvinar lateral of thalamus[148] **GMV:** Amygdala[45], Amygdala[46], Inferior parietal gyrus[66], Inferior temporal gyrus[93], Lobule X of cerebellar[112], Ventral anterior of thalamus[126]
All metrics for direct ANN model	**L.Hip:** Superior frontal gyrus[21], Posterior cingulate gyrus[39], Posterior cingulate gyrus[40], Cuneus[49], Cuneus[50];**R.Hip:** Superior frontal gyrus[21], Posterior cingulate gyrus[39], Cuneus[49], Cuneus[50], Superior occipital gyrus[53];**HE:** Posterior orbital gyrus[29], Hippocampus[41], Hippocampus[42], Ventral lateral of thalamus[127], Pulvinar lateral of thalamus[148];**GMV:** Posterior cingulate gyrus[39], Posterior cingulate gyrus[40], Amygdala[45], Amygdala[46], Putamen[78], Lenticular nucleus[80]	

Labels were: the abnormal brain region[the index number in the AAL3 atlas]; The maximum number of the abnormal brain regions for each metric was 6.

**Table 4:** The correlation between the clinical assessments and the prior three features in each metric.

The abnormal regions	MMSE	AVLT-ir	AVLT-dr	AVLT-r
L.Hip: Superior frontal gyrus[21]	0.33	0.33	0.40	0.35
L.Hip: Posterior cingulate gyrus[39]	0.35	0.32	0.45	0.33
L.Hip: Cuneus[49]	0.38	0.36	0.39	0.31
R.Hip: Superior frontal gyrus[21]	0.33	0.35	0.41	0.38
R.Hip: Cuneus[50]	0.40	0.36	0.37	0.33
R.Hip: Superior occipital gyrus[53]	0.35	0.47	0.43	0.43
HE: Hippocampus[41]	−0.40	−0.46	−0.47	−0.42
HE: Hippocampus[42]	−0.38	−0.44	−0.41	−0.36
HE: Posterior orbital gyrus[29]	0.41	0.36	0.45	0.32
GMV: Amygdala[46]	0.57	0.58	0.54	0.53
GMV: Amygdala[45]	0.48	0.52	0.46	0.45
GMV: Posterior cingulate gyrus[39]	0.48	0.45	0.41	0.42

### 3.3. Validation analysis results

By applying the ANN method to classify these three groups directly, a best accuracy of 73.81% was yielded, and the correct rates in AD, MCI and HC were 75.00, 63.64 and 84.48% respectively, and the detailed classification results of the direct three-class identification of the ANN method for all kinds of combinations of these four metrics were displayed in Table 5. In comparison, the naive Bayes classifier obtained a best accuracy of 72.62%, and the correct rates in AD, MCI and HC were 72.73, 65.15 and 81.03%. The RF method yielded a best accuracy of 73.21%, and the correct rates in AD, MCI and HC were 72.73, 63.64 and 84.48%. The classification performance of these two methods were relatively lower than or similar with the ANN method in direct three-class identification.

**Table 5:** The detailed classification performance of the direct three-class identification with ANN model.

Metrics	AD, MCI and HC based three classification performance				
	No. of features	AD	MCI	HC	Overall
L.HIP	44	47.73	46.97	67.24	54.17
R.HIP	28	54.55	54.55	58.62	55.95
HE	17	45.45	63.64	63.79	58.93
GMV	26	68.18	50.00	67.24	60.71
L.HIP+R.HIP	48	52.27	50.00	67.24	56.55
L.HIP + HE	24	40.91	63.64	72.41	60.71
L.HIP + GMV	17	75.00	63.64	68.97	68.45
R.HIP + HE	34	52.27	62.12	63.79	60.12
R.HIP + GMV	26	75.00	63.64	68.97	68.45
HE + GMV	25	72.73	63.64	84.48	73.21
L.HIP + R.HIP + HE	34	52.27	63.64	72.41	63.69
L.HIP + R.HIP + GMV	34	75.00	63.64	70.69	69.05
L.HIP + HE + GMV	25	72.73	63.64	84.48	73.21
R.HIP+ HE + GMV	25	72.73	63.64	84.48	73.21
L.HIP + R.HIP + HE + GMV	27	75.00	63.64	84.48	73.81

When down-sampling AD, MCI and HC patients into the same size of 44 subjects, the SVM based method obtained a best accuracy of 81.06% with all metrics, and the correct rates in AD, MCI and HC were 79.55, 77.27 and 86.36%, respectively. The ANN model obtained a best accuracy of 74.24%, and the correct rates in AD, MCI and HC were 75.00, 63.64, and 84.09%, respectively. The classification results with balanced samples were similar with those obtained on all subjects, demonstrating the stability of the classification performance.

When using the Brainnetome Atlas, the SVM based method yielded a best accuracy of 80.95% with all metrics, and the correct rates in AD, MCI and HC were 75.00, 81.82 and 84.48%, respectively. The ANN model obtained a best accuracy of 74.40%, and the correct rates in AD, MCI and HC were 75.00, 65.15 and 84.48%, respectively. The similar classification performance between these Brainnetome atlas and AAL3 atlas again indicated the feasibility of the AAL3 atlas in identifying AD, MCI and HC.

## 4. Discussion

The main aim of this work was to build effective methods for automatic identification of AD, MCI and HC subjects with multi-modal and multi-view metrics, and a best accuracy of 80.36% was achieved by simultaneously utilizing all four functional and structural metrics in the SVM based classification model. Correspondingly, the correct rates in AD, MCI and HC were 79.55, 78.79 and 82.76% respectively, indicating a powerful ability of multi-class diagnosis for AD and MCI patients. Additionally, when using the single modality metric, an optimal accuracy of 72.62% was achieved by using the HE metric in the SVM based method, and the correct rates in AD, MCI and HC were 56.82, 80.30 and 75.86% respectively, suggesting the HE metric could convey more useful and comprehensive information for the SVM based classification compared to other metrics.

To achieve a high classification performance for AD and MCI patients, several measures were taken for the classification models. Firstly, considering the fact that the multi-modal neuroimaging data have complementary information for each other (Hojjati et al., 2018; Zhou et al., 2019a,b), therefore both the sMRI and fMRI were adopted in this work to improve the diagnosis of AD and MCI. Through comparing the classification performance, we found the multi-modal metrics based identification models performed better than those with the single modality metric, which again validated the multi-modal imagings could enhance the classification performance. Secondly, when using all functional and structural characteristics of the multi-modal imagings, the feature dimension could reach up to 654 (164 × 4), but most of them are irrelevant and redundant for classification. Thus, the feature selection must be performed for dimensionality reduction to acquire a subset of optimal features (Pereira et al., 2009; Dai et al., 2012). In this paper, the MRMR algorithm, in combination with the SFC method, were utilized for feature selection, which significantly enhanced the classification performance in comparison to the classification model without feature selection. Actually, the best accuracy of the SVM based method for all the integrated features without feature selection was less than 50.00%, which was obviously lower than that with feature selection. It is worth mentioning that the feature selection was only constrained on the training data, which could avoid the potential overfitting. Thirdly, an inner 10-fold cross-validation was carried out to estimate the optimal modal parameters, which may improve the model generalization as well. Fourthly, the RBF kernel function, which can deal with the nonlinear problems between the features and labels, in combination with the grid search method for optimizing the parameters of the classification models were utilized in this work, which also has an important impact to promote the classification performance. In fact, we also tested the SVM based model with the linear kernel function for classifying AD, MCI and HC subjects in single modality or multi-modal conditions, and the overall correct rates were 60.12, 60.12, 70.24, 66.67 and 76.19%, by using the L.HIP based connectivity metric, the R.HIP based connectivity metric, the HE, the GMV and all the integrated metrics respectively, which were lower than those with the RBF kernel function. Fifthly, the AAL3 atlas replacing of the conventional AAL atlas was adopted to partition the whole brain cortex in this work, which provides more detailed parcellation for some brain areas. Actually, the AAL atlas was also tested for the multi-class diagnosis of AD, MCI and HC subjects with the SVM based classification model, and the best recognition rate was 73.21% by using all the features, which was lower than that with the newly proposed AAL3 atlas. Sixthly, two kinds of classification methods including the SVM based method and the ANN model were independently adopted to perform the multi-class identification of AD, MCI and HC subjects. Although the former method was intrinsically proposed for solving two-class classification problems, a three-class classification of AD, MCI and HC was well solved by applying the SVM method two times to deal with two kinds of binary classification problems in this work. The ANN method can handle a three-class classification problem with a direct identification or use two binary classifications instead, and our discrimination results demonstrated that the SVM based model performs better than these two kinds of ANN models, suggesting the importance of classifier selection in future studies. Currently, a direct three-class identification is still a big challenging problem, and several studies have indicated a relatively low classification accuracy in directly identifying AD, MCI and HC (Zhang et al., 2012; David et al., 2016; Liu et al., 2018). Thus, a direct three-class identification is not the primary aim of this work. Furthermore, a prior study has demonstrated that the multi-class SVM procedure can be carried out using a multiple trained binary SVM (Hussein et al., 2021), therefore two binary classifications with the SVM method were performed to identify AD, MCI and HC groups in this work, and the classification results demonstrated that the SVM based method performs better than the direct three-class algorithms including ANN and two other identification methods. Taken together, the proposed multi-modal metrics based SVM classification model with the newly proposed AAL3 atlas was effective in identifying the multi-class of AD, MCI and HC.

To validate the efficiency of the MRMR and SFC algorithms, the primary component analysis (PCA) was also tried for feature reduction in these two classification techniques with all the functional and structural metrics, and the cumulative variance contribution rate (CVCR) parameter of PCA was setting up ranging from 0.60 to 0.90 with a step-length of 0.05. In detail, the SVM model was firstly implemented to identify the mixed patients from the HC with different CVCR values. Then the second SVM based method was carried out only to the accurately identified AD and MCI cases under the optimal condition of the first SVM model. Overall, the SVM based method obtained a best accuracy of 79.17%, and the correct rates in AD, MCI and HC were 84.09, 71.21 and 84.48%, respectively. The direct ANN model yielded a best accuracy of 73.21% with the optimal CVCR of 0.80, and the correct rates in AD, MCI and HC subjects were 72.73, 63.64 and 84.48%, respectively. The slight superiority of the identification rates compared to the PCA algorithm demonstrated the efficiency of the MRMR and SFC.

In this paper, the overlapping abnormal brain regions were mainly located at posterior cingulate gyrus, superior frontal gyrus and cuneus in identifying the AD, MCI and HC, which were consistent with many prior studies that analyzed the functional or structural images with conventional univariate analysis in AD and MCI patients (Han et al., 2011; Marco et al., 2017). The posterior cingulate gyrus plays a prominent role in the default-mode network (DMN) that is significantly deficient in AD and MCI patients compared to HC (Wang et al., 2011; Dai et al., 2012). The superior frontal gyrus was detected with significant atrophy by utilizing the VBM analysis (Anne et al., 2007), and the cuneus was found with increased low-frequency blood oxygenation level dependent (BOLD) fluctuations in AD and MCI (He et al., 2007). All these brain regions were abnormal in different MRI metrics, suggesting the underlying mechanism of AD and MCI patients in these regions. Furthermore, some divergences of the detected abnormal brain regions in different metrics were also found in the SVM based model, including the HE abnormalities in the hippocampus, thalamus and the anterior orbital gyrus, the GMV abnormalities in amygdala, putamen and part cerebellar regions, the L.Hip based connectivity abnormalities in superior occipital gyrus and part vermis regions, and the R.Hip based connectivity abnormalities in superior occipital gyrus, inferior frontal gyrus, and part cerebellar and vermis regions. The main reason for the divergences may be attributed to the fact of the specificity in each modality and metric. The HE index reflects the persistent behaviors of the spontaneous brain activities, and even many investigators speculate that the HE reflects some inherent patterns of spontaneous discharge that could be adjusted by psychopathological and psychological conditions (Gentili et al., 2015; Long et al., 2018b). The GMV indicates the morphological information of the cortex, and the bilateral hippocampus based connectivity reflects the connection properties between the core region of hippocampus and other brain regions. The abnormalities of the structural and the functional metrics in above-mentioned regions are also important in the pathology of AD and MCI. Finally, a correlation analysis between the clinical assessments and the prior three abnormal regions obtained by directly applying the MRMR algorithm to these three groups in the ANN model, which were largely overlapping with the regions got in the SVM model, was also carried out. The significant correlation between clinical evaluations and all the abnormal regions also demonstrated the potential of these regions underlying AD and MCI patients.

Several limitations need to be discussed about this work. Firstly, some other brain atlases such as the Dosenbach atlas and the Desikan atlas have been proposed currently, which also could be utilized to investigate the abnormalities in AD and MCI patients. Because different atlases own different number of ROIs and different size of the regions, the differences of feature vectors due to different partitions would naturally affect the classification performance in the multivariate pattern analysis (Ota et al., 2014). Secondly, some other neuroimaging modalities such as the positron emission tomography (PET) and single photon emission computed tomography (SPECT), and other characteristic metrics such as the functional graph measures and the structural cortex thickness, also existed nowadays, and these modalities and characteristics could also be simultaneously used to differentiate AD and MCI patients form HC. At last, considering that the samples utilized in this work is not very large, thus we will further validate and verify the classification methods with a large size of samples obtained from a public database in future works.

## 5. Conclusion

In this paper, two classification techniques including the SVM and ANN methods were comparatively utilized to identify a multi-class of AD, MCI and HC by using single modal metric and multi-modal metrics, respectively. The results of the SVM and the ANN methods achieved the best accuracies of 80.36 and 74.40%, respectively, by using all multi-modal metrics, and the correct rates in AD, MCI and HC subjects were 79.55, 78.79 and 82.76%, respectively, in the SVM based classification model. Furthermore, the multi-modal metrics based identification models performed better than those with the single modality. Therefore, the classification methods utilized in this work could be successfully implemented in identifying AD, MCI and HC, and different modalities could offer more useful information for classification compared to single modality.

## Data availability statement

The raw data supporting the conclusions of this article will be made available by the authors, without undue reservation.

## Ethics statement

The studies involving humans were approved by Nanfang Hospital affiliated to Southern Medical University. The studies were conducted in accordance with the local legislation and institutional requirements. The participants provided their written informed consent to participate in this study.

## Author contributions

ZL and JL made substantial contributions to the conception, design, analysis and interpretation of data and drafted the manuscript. ZL, JL, JF, BL, YD, SQ, JC, JY, and BJ made contributions to the revision of the manuscript. JM, ZL, and BJ made contributions to the data acquisition. BJ made contributions to conception and interpretation of data, and determined the final version to be submitted for publishing. All authors contributed to the article and approved the submitted version.

## Funding

The work was supported by open fund project of Beijing Key Laboratory of Fundamental Research on Biomechanics in Clinical Application (2023KF05).

## Conflict of interest

The authors declare that the research was conducted in the absence of any commercial or financial relationships that could be construed as a potential conflict of interest.

## Publisher’s note

All claims expressed in this article are solely those of the authors and do not necessarily represent those of their affiliated organizations, or those of the publisher, the editors and the reviewers. Any product that may be evaluated in this article, or claim that may be made by its manufacturer, is not guaranteed or endorsed by the publisher.

## Footnotes

^1^ http://www.cs.ucl.ac.uk/staff/G.Ridgway/vbm/get_totals.m
