# Supplementary material for: Identifying Alzheimer’s disease and mild cognitive impairment with atlas-based multi-modal metrics
Source: Front Aging Neurosci. 2023 Aug 31;15:1212275. doi: 10.3389/fnagi.2023.1212275 (PMC10501142; doi:10.3389/fnagi.2023.1212275)
Supplement: Supplementary file 1 [file Data_Sheet_1.PDF]

Table 1: The selected features in each fold of external 10-fold cross-validation in the SVM based model in identifying the mixed AD and MCI from HC subjects with all metrics.

Fold	No. of features	The index numbers of abnormal regions in AAL3 for each metric
1	17	L.Hip: [7], [39], [53], [113], [120]<br>R.Hip: [10], [53]<br>HE: [29], [41], [113], [156]<br>GMV: [39], [40], [45], [46], [77], [112]
2	17	L.Hip: [7], [39], [120], [159]<br>R.Hip: [10], [31], [53]<br>HE: [29], [41], [123], [156]<br>GMV: [39], [40], [45], [46], [69], [77]
3	17	L.Hip: [7], [39], [113]<br>R.Hip: [10], [17], [53]<br>HE: [27], [29], [41], [113], [156]<br>GMV: [39],[40],[46],[69],[77],[112]
4	16	L.Hip: [39],[113]<br>R.Hip: [10],[53],[165]<br>HE: [27],[41],[119],[123],[156]<br>GMV: [39], [40], [45], [46], [69], [112]
5	18	L.Hip: [7], [39], [53], [113]<br>R.Hip: [7], [10], [17], [39], [53]<br>HE: [29], [41], [113]<br>GMV: [39], [40], [45], [46], [69], [77]
6	15	L.Hip: [39], [113], [120]<br>R.Hip: [10], [17], [31], [53]<br>HE: [29], [41], [156]<br>GMV: [39], [40], [45], [69], [77]
7	16	L.Hip: [7], [39], [53]<br>R.Hip: [10], [17], [53]<br>HE: [27], [29], [41], [156]<br>GMV: [39], [40], [45], [46], [69], [77]
8	17	L.Hip: [7], [39], [113]<br>R.Hip: [7], [10], [31], [53]<br>HE: [29], [41], [119], [156]<br>GMV: [39], [40], [45], [46], [69], [77]
9	15	L.Hip: [7], [53], [113], [120]<br>R.Hip:[10], [17]<br>HE: [29], [41], [123]<br>GMV: [39], [40], [45], [46], [77], [112]
10	16	L.Hip: [7], [39], [113], [159]<br>R.Hip: [10], [53], [165]<br>HE: [27], [41], [113], [156]<br>GMV: [39], [46], [69], [77], [112]

Of note: the detailed names can refer to this publication (Rolls et al., 2020).

Table 2: The retained features in each fold of external 10-fold cross-validation in the SVM based model in identifying AD and MCI patients with all metrics.

Fold	No. of features	The index numbers of abnormal regions in AAL3 for each metric
1	17	L.Hip: [7], [21], [39], [53], [113]<br>R.Hip: [18], [50], [124], [113]<br>HE: [27], [147], [148]<br>GMV:[46], [66], [93], [112], [126]
2	16	L.Hip: [7], [39], [105], [113]<br>R.Hip: [18], [50], [124], [106]<br>HE: [147], [148]<br>GMV:[45], [46], [66], [93], [120], [126]
3	15	L.Hip: [39], [113]<br>R.Hip: [18], [50], [124], [113]<br>HE: [136], [147], [148]<br>GMV:[45], [46], [66], [93], [112], [126]
4	16	L.Hip: [7], [53], [113], [147]<br>R.Hip:[50], [124], [106]<br>HE: [136], [147], [148]<br>GMV:[45], [46], [93], [112], [120], [126]
5	16	L.Hip: [7],[21],[39],[113]<br>R.Hip: [18],[50],[124],[106]<br>HE: [147], [148]<br>GMV:[45],[46],[66],[93],[112],[126]
6	14	L.Hip: [7], [39], [105]<br>R.Hip: [18], [50], [124], [120]<br>HE: [147], [148]<br>GMV:[45], [66], [112], [120], [126]
7	15	L.Hip: [7], [39], [113]<br>R.Hip: [18], [50], [124], [120]<br>HE: [136], [148]<br>GMV:[45], [46], [66], [93], [120], [126]
8	15	L.Hip: [21], [39], [113]<br>R.Hip: [18], [124], [113]<br>HE: [105], [147], [148]<br>GMV:[45], [46], [66], [93], [112], [126]
9	15	L.Hip: [7], [39], [113], [169]<br>R.Hip: [18], [50], [124]<br>HE: [147], [148]<br>GMV:[45], [46], [66], [93], [112], [126]
10	16	L.Hip: [7], [21], [39], [113]<br>R.Hip: [18], [50]<br>HE: [27], [105], [147], [148]<br>GMV:[45], [46], [66], [93], [112], [126]
